# Protocols for dry DNA storage and shipment at room temperature

**DOI:** 10.1111/1755-0998.12134

**Published:** 2013-06-24

**Authors:** Natalia V Ivanova, Masha L Kuzmina

**Affiliations:** Biodiversity Institute of Ontario, University of GuelphGuelph, ON, N1G 2W1, Canada

**Keywords:** Biomatrica, DNA preservation, dry storage, PVA, trehalose

## Abstract

The globalization of DNA barcoding will require core analytical facilities to develop cost-effective, efficient protocols for the shipment and archival storage of DNA extracts and PCR products. We evaluated three dry-state DNA stabilization systems: commercial Biomatrica® DNAstable® plates, home-made trehalose and polyvinyl alcohol (PVA) plates on 96-well panels of insect DNA stored at 56 °C and at room temperature. Controls included unprotected samples that were stored dry at room temperature and at 56 °C, and diluted samples held at 4 °C and at −20 °C. PCR and selective sequencing were performed over a 4-year interval to test the condition of DNA extracts. Biomatrica® provided better protection of DNA at 56 °C and at room temperature than trehalose and PVA, especially for diluted samples. PVA was the second best protectant after Biomatrica® at room temperature, whereas trehalose was the second best protectant at 56 °C. In spite of lower PCR success, the DNA stored at −20 °C yielded longer sequence reads and stronger signal, indicating that temperature is a crucial factor for DNA quality which has to be considered especially for long-term storage. Although it is premature to advocate a transition to DNA storage at room temperature, dry storage provides an additional layer of security for frozen samples, protecting them from degradation in the event of freezer failure. All three forms of DNA preservation enable shipment of dry DNA and PCR products between barcoding facilities.

## Introduction

The globalization of DNA barcoding will require core analytical facilities to develop cost-effective, efficient protocols for the shipment and archival storage of DNA extracts and PCR products. DNA barcoding uses short sections of DNA from a standardized region of the genome for species identification and discovery; a 648 base-pair region in the mitochondrial cytochrome c oxidase 1 gene (CO1) is being used for most animal groups (Hebert *et al*. 2003). The ideal DNA preservation system should work on diluted samples and allow single-step recovery for subsequent PCR (650–800 bp) or sequencing reactions.

We evaluated three dry-state DNA stabilization systems, one commercial and two home-made, on 96-well panels of insect DNA stored at 56 °C and at room temperature. All tested approaches rely on protection of DNA in a dry state in the presence of protective agents.

Trehalose is a well-known agent for cryopreservation and lyophilization of biological samples (Taylor *et al*. 1994; McGinnis *et al*. 2005) diluted DNA (Smith & Morin 2005; Zhu *et al*. 2007) and RNA for use in vaccine studies (Jones *et al*. 2007). Many invertebrates undergoing anhydrobiosis, such as brine shrimp or tardigrades, often produce the sugar trehalose (Crowe 2008). Among the most commonly used disaccharides (sucrose and trehalose), trehalose is preferable for stabilization of biomolecules due to its higher glass transition temperature. Trehalose stabilizes DNA due to its ability to form tight hydrogen bonds to the phosphate groups, which leads to shielding of the large phosphate–phosphate repulsion. Trehalose also probably interacts with other polar groups of DNA, which, when combined with hydrogen bonds to phosphate groups, makes trehalose a water-like solvent for DNA and stabilizes the base stacking during and after dehydration (Zhu *et al*. 2007).

The advantages of trehalose can be summarized as follows: (i) more flexible formation of hydrogen bonds with proteins and DNA due to the absence of internal hydrogen bonds; (ii) less hygroscopicity; (iii) low chemical reactivity; and (iv) prevention of water plasticizing the amorphous phase partly by forming trehalose–protein–water microcrystals (Crowe *et al*. 1992; Librizzi *et al*. 1999).

Polyvinyl alcohol (PVA) is a polymer of great interest because of its many desirable characteristics specifically for various biomedical and pharmaceutical applications (Hassan *et al*. 2000). PVA is widely used in forensics for sampling of gunshot residues and includes the advantage of embedding all particles including bloodstains. It has been shown that blood traces embedded in PVA film can be successfully used for PCR typing analysis (Schyma *et al*. 1999). Studies of PVA–DNA cryogel membranes have demonstrated strong interactions between DNA and PVA (Papancea *et al*. 2008). The hydrogen bonding between DNA and PVA enables preparation of PVA–DNA nanoparticles suitable for gene and protein delivery applications (Kimura *et al*. 2004; Liao *et al*. 2005). Besides these unique characteristics, PVA is not toxic, does not affect enzymatic reactions, and keeps some amount of water molecules tightly attached even in dehydrative solvents, which is advantageous for enzyme stabilization (Szczęsna-Antczak *et al*. 2002). All these properties make PVA an ideal candidate for DNA preservation.

Recently, new room temperature storage technologies relying on synthetic preservation matrices have been developed by Biomatrica Inc. Although they have been tested on human DNA (Hernandez *et al*. 2009; Wan *et al*. 2010; Frippiat *et al*. 2011; Lee *et al*. 2012), there is virtually no data on the performance of these systems with invertebrate DNA. In the present study, we evaluated comparative performance of trehalose, Tris–HCL buffered PVA and Biomatrica's DNAstable on insect DNA in a 4-year storage experiment.

## Materials and methods

### DNA extraction

Due to limited DNA yield from a medium-size insect, we decided that it was more valuable to explore as many experimental conditions and time points as possible rather than using traditional replicates in our experiment design. We used one or two individuals from nine medium-size Lepidoptera species (Table 1). Thus, the twelve specimens serve as replicates for each treatment. Each DNA extract was tested for eight dilutions and all treatments.

**Table 1:** Samples used to prepare DNA storage panels

	Well locators–serial dilution	Species
Sample 1	1 (H-A)	*Colocasia propinquilinea*
Sample 2	2 (H-A)	*Estigmene acrea*
Sample 3	3 (H-A)	*Mythimna unipuncta*
Sample 4	4 (H-A)	*Noctua pronuba*
Sample 5	5 (H-A)	*Noctua pronuba*
Sample 6	6 (H-A)	*Spilosoma dubia*
Sample 7	7 (H-A)	*Spilosoma dubia*
Sample 8	8 (H-A)	*Euclidia cuspidea*
Sample 9	9 (H-A)	*Euclidia cuspidea*
Sample 10	10 (H-A)	*Crocigrapha normani*
Sample 11	11 (H-A)	*Caripeta piniata*
Sample 12	12 (H-A)	*Tetracis crocallata*

The abdomens were removed, and three legs and three wings of each specimen were used for DNA extraction with two silica-based extraction methods, hereafter named MN and GF: a commercial kit NucleoSpin 96 (Macherey-Nagel GmbH & Co. KG, Düren, Germany) and the standard Canadian Centre for DNA Barcoding (CCDB) DNA extraction protocol using a glass fibre plate (Ivanova *et al*. 2006) following corresponding instructions with minor modifications.

One-thousand-nine-hundred microlitres of lysis buffer (Insect Lysis Buffer or T1) with Proteinase K (Life Technologies; Invitrogen, Carlsbad, NM, USA) was added to each sample, which was ground with a pestle inside the tube and incubated overnight at 56 °C. Two-hundred-microlitre aliquots of each lysate were transferred into Ultident tube racks, mixed with 400 μL of corresponding binding buffer and transferred to a binding plate [tissue binding plate in MN kit or 3 μm GF over 0.2 μm Bioinert membrane Acroprep plate (Pall Life Sciences, Ann Arbor, MI, USA)]. Plates were centrifuged at 6000 ***g***. The first wash volume consisted of 400 μL, followed by a second wash of 750 μL using the corresponding buffer systems. Plates were incubated at 56 °C for 30 min, and DNA was eluted with 100 μL of ddH_2_O preheated to 56 °C. After extraction, the eluates belonging to the same sample were combined into a single tube.

### Serial dilution

Hereafter, two DNA panels will be referred as GF and MN (same as extraction protocols). Unfortunately, the GF DNA extracted with standard CCDB extraction protocol could not be accurately quantified using Nanodrop at the time of experiment set-up, due to carryover of chaotropic salts and Triton X-100 (Ivanova *et al*. 2008), while MN DNA concentration was below accurate detection with Nanodrop; therefore, we used Qubit fluorometer with HS DNA assay (Life Technologies, Invitrogen) to measure undiluted DNA stocks after 4 years of storage at −20 °C. Starting average DNA concentration was 0.1 ng/μL for MN DNA and 1.8 ng/μL for GF DNA. Typical concentration of insect DNA extracted at the CCDB from medium-size insect legs is around 1.5–4 ng/μL, which is corresponding to GF DNA concentration range.

Each DNA sample was aliquoted undiluted into row H, and diluted 2× (row G), 4× (row F), 8× (row E), 10× (row D), 20× (row C), 50× (row B) and 100× (row A) in a PROgene Mini Tube System (Ultident Scientific, St. Laurent, QC, Canada). As a result, we created two DNA panels containing 400 μL in each well. Each panel was then used to test different treatments and storage conditions (Table 2).

**Table 2:** Experiment set-up

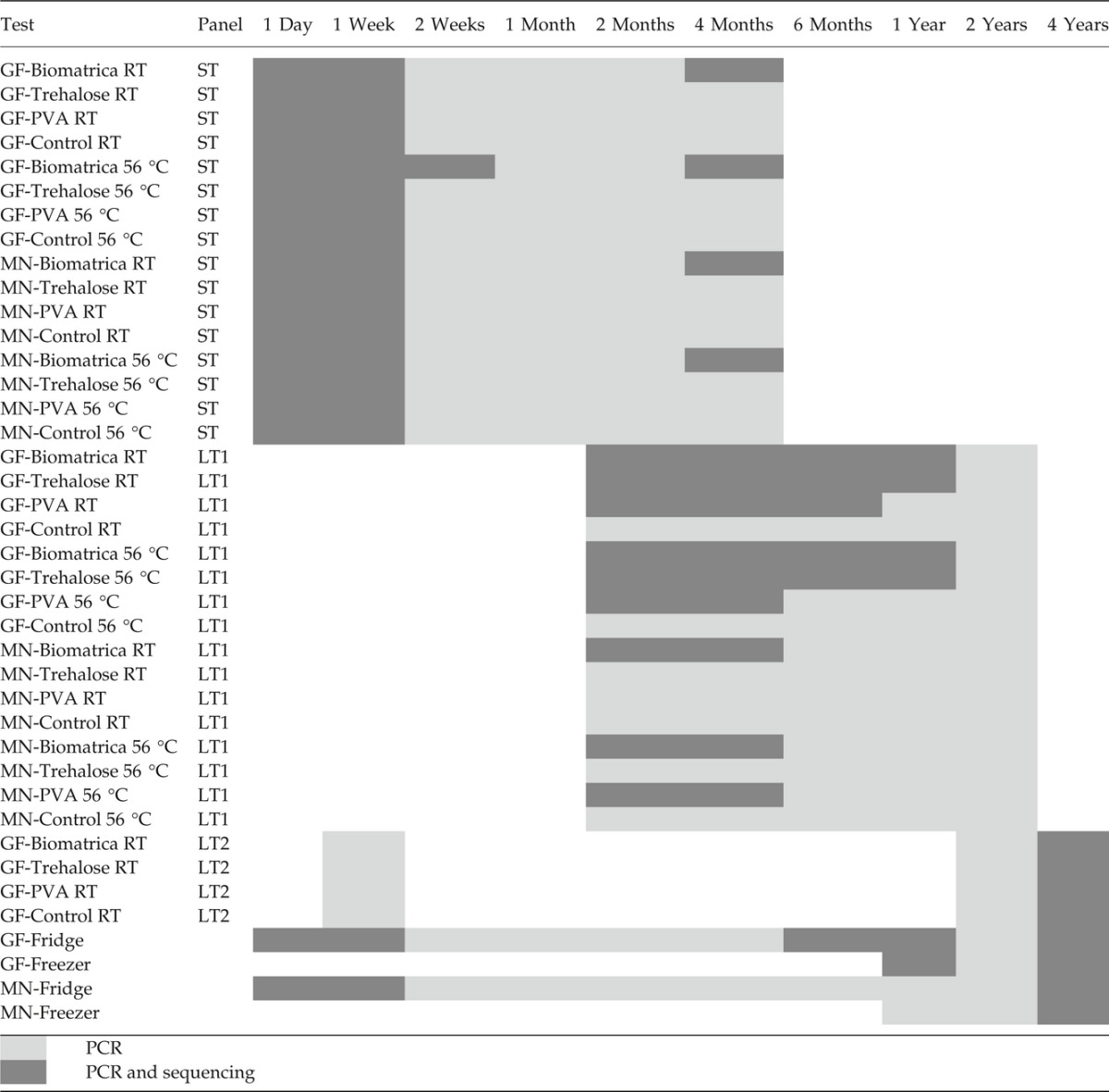

### Short-term storage panels

Skirted twin.tec 96-well plates (Eppendorf, Hamburg, Germany) were used for storage. We applied 16 μL of DNA from each panel to a DNAstable Biomatrica plate (Biomatrica Inc., San Diego, CA, USA), a plate with 5 μL of 10% trehalose (Cat. #90210; Sigma-Aldrich, St. Louis, MO, USA), a plate with 5 μL of 1% PVA (Cat. No P8136, Sigma-Aldrich), 200 mm Tris–HCl, pH 8.0 and to an empty plate as a control. Plates were dried overnight at 56 °C or at RT (room temperature), covered with aluminium foil, and stored at 56 °C or at room temperature. DNA recovery was tested after 1 day, 1, 2 weeks and 1, 2 and 4 months (ST panel–Table 2).

### Long-term storage panels

1. A separate aliquot of 10 μL was applied to a Biomatrica plate, a plate with 5 μL of 10% trehalose, a plate with 5 μL of 1% PVA, 200 mm Tris–HCl, pH 8.0 and to an empty plate as a control and tested as above after 4, 6 months, 1 and 2 years of storage at 56 °C and RT (LT1 panel–Table 2).
2. Another aliquot of 26 μL of the GF panel was applied to a Biomatrica plate, a plate with 5 μL of 10% trehalose, a plate with 5 μL of 1% PVA, 200 mm Tris–HCl, pH 8.0 and to an empty plate as a control and tested as above after 1 week, 2 and 4 years of storage at RT (LT2 panel–Table 2).
3. Fridge storage: an aliquot of each panel was transferred to a clean 96-well plate, covered with foil, and stored at 4 °C with no additives (tested at 1 day, 1, 2 weeks, 1, 2, 4, 6 months, 1, 2 and 4 years).
4. Freezer storage: original serial dilutions were stored in tube racks at −20 °C. These aliquots underwent freeze-thaw cycles at each time point during testing (tested at 1, 2 and 4 years).

At each time point, DNA was resuspended in water and an aliquot of 2 μL was taken for PCR. The resuspension volumes were adjusted for each time point, so the DNA concentrations remained constant (e.g. 16, 14, 12, 10 μL). The leftovers were dried as described above.

### PCR and sequencing

Each PCR reaction contained a total volume of 12.5 μL consisting of 5% trehalose [D-(+)-Trehalose dehydrate], 1.25 μL of 10× reaction buffer (Life Technologies, Invitrogen), 2.5 mm of MgCl_2_, 1.25 pmol each of forward and reverse primer, 50 μm of dNTP, 0.3 U of Platinum Taq DNA polymerase (Life Technologies, Invitrogen) and 2 μL of DNA template. The thermocycle consisted of 94 °C for 1 min, five cycles of 94 °C for 40 s, 45 °C for 40 s and 72 °C for 1 min, followed by 35 cycles of 94 °C for 40 s, 51 °C for 40 s and 72 °C for 1 min, with a final extension at 72 °C for 5 min (Hajibabaei *et al*. 2005; deWaard *et al*. 2008).

PCR products were visualized on 2% E-gel 96 system (Life Technologies, Invitrogen). Wells containing bands were counted as positive, with no differentiation between strong and weak bands. To validate PCR success, selected treatments were sequenced as described in Ivanova & Grainger (2007). Sequencing reactions were analysed on 3730xl DNA Analyzer (Life Technologies, Applied Biosystems) following manufacturer's instructions.

### Data analysis and visualization

Selected sequences were analysed using CodonCode Aligner (CodonCode Corporation, Centerville, MA, USA) to screen for possible cross-contamination and to confirm authenticity.

PCR success was calculated as percentage of positive wells per plate (96 samples), per sample (8 wells in a column) or per dilution (12 wells in a row); sequencing success was calculated as percentage of sequences >500 bp (per sample). Contiguous read length (CRL) and average raw signal intensity (ARSI) distribution statistics were retrieved from raw sequence data using Sequence Scanner 1.0 (Applied Biosystems). The following cut-offs were used to filter failed or poor-quality sequences: 500 bp for CRL and 150 ARSI for signal strength. ARSI was calculated for each sample based on ARSIs of individual bases ([ARSI (A)]+[ARSI (C)]+[ARSI (G)]+[ARSI (T)])/4.

Resulting output reports were copied to Microsoft Excel 2010 (Microsoft Corporation, Redmond, USA), annotated and analysed using Tableau software 7.0 (Tableau Software Inc., Seattle, WA, USA).

## Results and discussion

**Fig 1:**
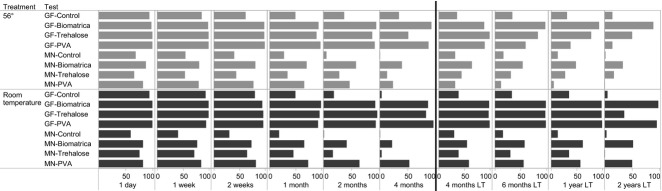
Degradation dynamics at 56 degrees and room temperature for ST panel tested after 1 day to 4 months and LT1 panel tested after 4 months to 2 years (separated by black line) measured as PCR success (%) per plate.

The results of our DNA storage experiment can be represented as an interaction of several independent factors affecting DNA preservation, such as concentration, protective agent, temperature and number of resuspension events, projected onto a timescale (Fig. 1).

Diluted DNA samples have been often used as a model in DNA preservation experiments (Smith & Morin 2005) to reflect the constant rate of DNA degradation. Because concentrated samples have more long DNA molecules to start with, it takes longer for all of them to drop under the minimum size of template needed for the PCR. In our experiment, the PCR success for diluted DNA (MN) was lower in both short and long storage experiments, compared to results with concentrated DNA (GF). Thus, MN demonstrated faster response to storage conditions. For example, unprotected MN degraded completely after 2 months of short-term storage at RT, whereas it took 2 years for unprotected GF to reach almost complete degradation under the same conditions (Fig. 1). Unprotected MN-panel completely degraded at RT after the 5th resuspension event at RT and after the 6th resuspension event at 56 °C in the short-term storage experiment. It took 2 years and four resuspension events to observe obvious DNA degradation (35% PCR success) for the concentrated panel (GF) in trehalose at room temperature, compared with 1 month of storage and four resuspension events for diluted (MN) DNA (46% PCR success).

There was a clear difference between the three different protecting agents. The Biomatrica was the best preserving agent in all storage experiments, except for the short-term storage at room temperature, where PVA showed better PCR success after the 6th resuspension event for both panels (53% vs. 23% for MN and 96% vs. 86% for GF).

**Fig 2:**
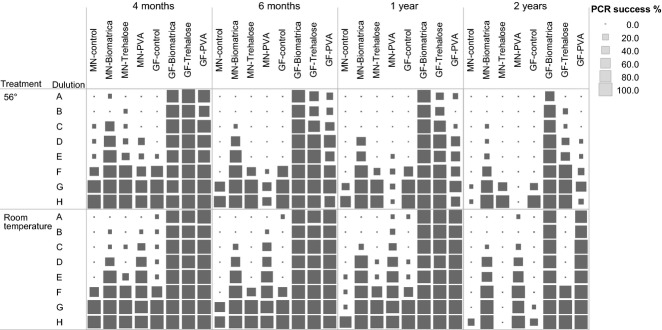
Degradation dynamics of various dilutions (A – 100×, B – 50×, C – 20×, D – 10×, E – 8×, F – 4×, G – 2×, H – undiluted DNA) for LT1 panel at 56 °C and room temperature, measured as PCR success (%) per dilution.

Within each panel and each extraction method, diluted samples (dilutions G-A) always degraded faster, especially for unprotected DNA. Figure 2 shows degradation dynamics of dilutions in LT1 panel from 6 months to 2 years of storage. Having lower starting DNA concentration, MN panels showed faster degradation rate even for undiluted DNA (dilution H).

After 2 years of storage, the second best preserving agent at room temperature was Tris-buffered PVA, which provided adequate protection (95% for GF and 50% for MN) at room temperature but was not as efficient at 56 °C (15% for GF and complete degradation for MN; Fig. 1). Trehalose was the second best protectant after Biomatrica at 56 °C (50% for GF and 17% for MN), but, contrary to PVA, did not offer good protection at room temperature (35% for GF and complete degradation for MN).

Interestingly, storage at 56 °C, which was introduced as part of the experiment to emulate accelerated ageing, showed unexpectedly higher PCR success compared with RT, especially for unprotected DNA and DNA protected with trehalose, in both short and long storage experiments. This was presumably due to more stable humidity conditions at 56 °C, compared with RT. Unprotected DNA in both panels was degrading faster at room temperature compared with 56 °C, as indicated by 3% PCR success at RT and 35% at 56 °C for GF-control after 4 months and 6 resuspension events (Fig. 1). Oppositely, higher temperature dramatically decreased PCR success for PVA for both MN and GF panels, in comparison with RT.

Overall, the resuspension events were a critical factor contributing to DNA degradation (compare left and right part of Fig. 1 separated by a black line). In a short 4-month storage experiment, panels protected with trehalose were most vulnerable to the number of resuspensions (both MN and GF). The unprotected diluted panel reached same level of degradation after 2 months and the 5th resuspension event and after 2 years and the 4th resuspension event as indicated by low PCR success (0% and 3% PCR success, respectively). The degradation of the Biomatrica panel during multiple resuspensions was slower compared with trehalose, and the PVA showed the least sensitivity to this factor. The goal of our study was to explore the limits of unpredictable temperature and humidity fluctuations, which samples might encounter during preparation, storage, shipment and handling. Therefore, we did not use any desiccants or special equipment, such as a DNA concentrator, for DNA drying at room temperature. In a recent study on DNA preservation of forensic samples in Biomatrica sample matrix at room temperature (Lee *et al*. 2012), samples were stored with or without desiccants. Similar to our findings, samples preserved without desiccants degraded faster. The accelerated rate of DNA degradation after multiple resuspension events observed in our experiment could be caused by fluctuations in humidity and exposure of DNA to air during storage, drying and resuspension, which is in concordance with the findings of Colotte *et al*. (2011).

In all long-term storage experiments, Biomatrica was the better protectant, compared with PVA and trehalose. After 2 years of storage and four resuspensions, Biomatrica provided the best protection at 56 °C and at room temperature. The PCR success rates for GF and MN panels consisted of 97% and 52% for room temperature and 89% and 33% for 56 °C.

The second best preserving agent at room temperature was Tris-buffered PVA, which provided adequate protection (95% for GF and 50% MN) at room temperature but was not as efficient at 56 °C (13% for GF and complete degradation for MN). Trehalose was the second best protectant after Biomatrica at 56 °C (50% for GF and 17% for MN), but, contrary to PVA, did not offer good protection at room temperature (35% for GF and complete degradation for MN).

**Fig 3:**
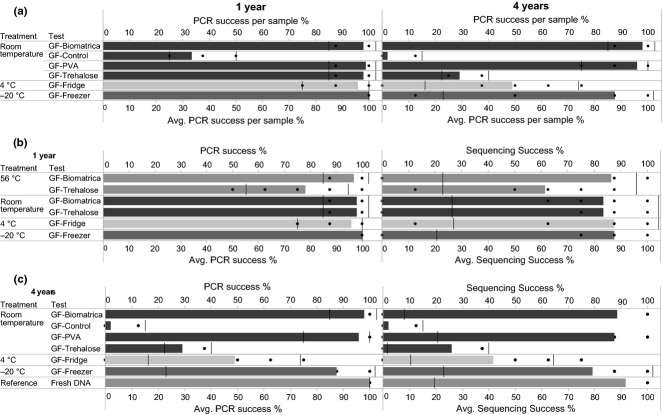
(a) Average PCR success per sample (%) on concentrated panels after 1 and 4 years of storage at room temperature in comparison with fridge and freezer (3rd resuspension event for each panel); (b) average PCR and sequencing success (%) after 1 year of storage and three resuspension events for selected treatments; (c) Average PCR and sequencing success (%) after 4 years of storage and three resuspension events for selected treatments compared with fresh DNA data used as a reference point. Black dots indicate percentage of dilutions that amplified or sequenced for each of 12 samples (one dot may represent more than one sample); black lines indicate standard deviation (± one).

Figure 3a shows comparison of the 3rd resuspension event after 1 and 4 years of storage of concentrated panel at room temperature. After 1 year, there was no significant difference in average PCR success per sample between different treatments (97–100%), except for nonprotected DNA (33%). After 4 years at room temperature (with three resuspension events), the best protection was observed in Biomatrica (98%), followed by PVA (96%) and freezer storage (−20 °C; 88%). Serious DNA degradation was observed in nonprotected DNA (2%), trehalose (29%) and fridge storage (4 °C; 49%).

To validate PCR success, selected treatments preserved at RT and 56 °C were sequenced. The sequencing success correlated with PCR success (Fig. 3b,c). However, Sample 10 failed to produce readable sequence in all treatments including freshly extracted DNA, hence causing proportionally lower sequencing success for the concentrated panel. After 4 years of storage, the PCR and sequencing success were higher for Biomatrica and PVA treatments in comparison with freezer storage.

**Fig 4:**
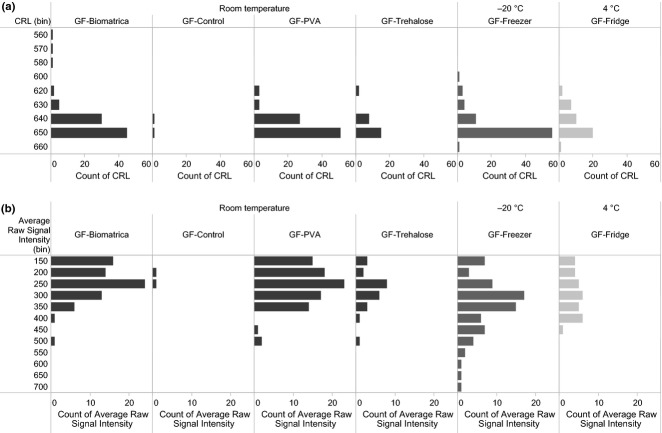
(a) Contiguous read length distribution after 4 years of storage (500 bp was used as cut-off criteria to filter failed sequences); (b) Average raw signal intensity distribution after 4 years of storage (150 rfu was used as cut-off criteria to filter background noise and failed sequences).

Based on CO1 DNA Barcode standards (Ratnasingham & Hebert 2007), we used 500 bp to evaluate sequencing success as a cut-off value. As an additional measure of DNA preservation, we evaluated distribution of CRL and ARSI (Fig. 4). In spite of lower PCR and sequencing success, DNA stored in the freezer showed the highest number of long reads >650 bp (57 for freezer vs. 45 for Biomatrica and 51 for PVA; Fig. 4a) and the highest number of samples with average raw signal intensity >150 rfu (63 samples for freezer vs. 57 for PVA and 45 for Biomatrica; Fig. 4b), which might be an indication of better DNA integrity in frozen samples.

Storing DNA in a dry state in the presence of trehalose (Smith & Morin 2005) or other kind of protective matrix such as Biomatrica's DNAstable can add an additional layer of stability for storage at −80 °C and is recommended as a standard procedure for DNA preservation (Knebelsberger & Stöger 2012). While Biomatrica's proprietary matrix offers the best protection, both trehalose and PVA can be successfully utilized as cost-efficient alternatives. All three storage media can be successfully utilized for shipment and exchange of PCR products and DNA between DNA barcoding facilities.

## Conclusions

Overall, the dry storage media (trehalose, Biomatrica or PVA) provided sufficient protection for short-term storage of DNA at room temperature, thus enabling shipment and exchange of DNA extracts and PCR products between DNA barcoding facilities. Although trehalose might be sufficient for short-term storage, we recommend Biomatrica and Tris-buffered PVA for long-term storage and for sample exchange with tropical countries. Desiccants should be used to minimize exposure to humidity. Our experiments indicate that temperature was a crucial factor for DNA quality which has to be considered especially for long-term storage. Therefore, it is premature to advocate transition to DNA storage at room temperature.

## Notes

N.V.I. designed the experiment, performed the experiment and analysed data, M.L.K. performed the experiment, both authors contributed equally to the manuscript.

## Data Accessibility

Original images of E-gels, sequence trace data and MS Excel file used for calculations in Tableau were archived at DRYAD entry doi:10.5061/dryad.6p2h0 (http://datadryad.org).
